# Sound localization in web-based 3D environments

**DOI:** 10.1038/s41598-022-15931-y

**Published:** 2022-07-15

**Authors:** Chinmay Rajguru, Giada Brianza, Gianluca Memoli

**Affiliations:** School of Engineering and Informatics, University of Sussex, Falmer, Brighton, BN1 9RH UK

**Keywords:** Psychology, Engineering

## Abstract

Sound delivery is a key aspect of immersivity in virtual and augmented reality (VR/AR), with studies hinting at a correlation between users’ ability to locate sounds around them and the ‘feeling of being there’. This is particularly true for WebVR, a method of delivering immersive experiences through a local web browser that has recently captured attention in multiple industries. In WebVR, audio is the main spatial cue. Designers need to select the correct number of sound sources so that users perceive the location of incoming sound correctly. Information on how users localize sound is essential. Sound localization experiments, so far, have been run only in empty spaces or closed rooms, without clear indications for designers in WebVR. Thus, in this study, we investigate sound localization directly through WebVR. To do so, we designed a traditional empty room for training and a city-like virtual environment for testing purposes. In our paper, we also discuss key design parameters, differences in perception for vertical and horizontal directions, the impact of training, and the role of changing virtual environments. In addition, we introduce and test a new sound cue along with the traditional pink noise sound to measure and explore the impact of different sound cues in different environments. The results demonstrate the potential of exploring sound localization using WebVR, and our study will support the development of virtual experiences in human-computer interaction that may be able to reach a large number of participants using a local web browser.

## Introduction

Audio cues are crucial enablers both in virtual reality (VR) and augmented reality (AR). This is because audio cues underpin ‘immersivity’, which is the third component of successful immersive virtual environments (IVEs)^1^, wherever they sit across the ‘reality-virtuality continuum’^2^. Audio cues are instrumental to the suspension of disbelief necessary to create full immersion in virtual experiences and, in particular, to the ‘feeling of being there’^1,3^.

This, in turn, depends not only on where sound sources are located by the programmer (e.g., using Unity or Unreal engine), but also on how it is delivered—e.g. through headphones or speaker arrays^4,5^—and on the accuracy with which the users can actually localise the different sources.

Sound design for IVEs is, therefore, a user-centered process^6^, but very little research is available to determine how users perceive audio cues in IVEs. Results on sound localisation (i.e. the psychoacoustic ability of end-users to localize sound cues in a 3D environment) and minimum audible angle (the *M*AA is the physiological limit at which a user can distinguish two different sources, typically reported as $2^{\circ }$) are simply transferred to IVEs from studies run either in real room environments or in virtual closed rooms, with typically less than 20 participants^7–9^. However, in a context where new technologies (e.g. low-cost VR/AR headsets and even smartphones) are bringing mixed realities to a multitude of new users^10,11^, this lack of direct knowledge causes a discrepancy between what can be programmed (by the designer) and what is eventually perceived (by the user). It is, therefore, crucial to establish protocols to run localisation experiments directly in virtual environments.

In this study, we explore the possibility of employing WebVR as a platform for sound localization experiments, highlighting the benefits and shortcomings of doing so. We, therefore, adapted the classical protocol of a sound localization experiment to ‘WebVR’. Here, WebVR describes 3D virtual experiences delivered using a web browser on a desktop or a laptop, in a platform-independent way. It is therefore not surprising that numerous user experience designers, artists, and developers have recently shown interest in this technology, with well-known companies such as Google^12^ and Mozilla^13^ also encouraging content creation for WebVR platforms since it can be accessed easily, shared widely, and effectively enjoyed from anywhere in the world. The use of web-based 3D environments, another term for WebVR, quickly gained momentum due to the recent pandemic and is currently the most used portal to IVEs^14^. We selected WebVR over other technologies because of its lack of user movement: the typical user sits in front of a computer screen, interacting with the virtual environment (VE) while listening to sound through headphones (in-ear/over-ear). In WebVR, audio is the main spatial cue and its effect is magnified. In addition, we were also interested to know if we can make this method easily available to many users remotely who are not necessarily experts in acoustics.

Through an online user study with 210 participants, we used our protocol to evaluate the impact on localisation accuracy of binary parameters like the virtual environment (‘empty room’ and ‘cityscape’), the sound cue (the classical ‘pink noise’ and a more familiar ‘cycle bell’), and the training time (‘short’ and ‘long’), finding that there are optimal conditions for sound localisation in IVEs. Once our protocol was established, we used it to examine the potential impact of cross-modal relationships between visual and audio on psycho-acoustic judgments^15,16^, finding that user expectations may be the main factor underpinning these effects. We also highlighted the impact of ‘non-acoustic’ factors, like self-assessed sensitivity or participant age: two aspects neglected in previous localisation experiments, but known as the most relevant ‘non-acoustic factors’ in other fields (e.g., soundscape research). Finally, we established that the accuracy follows a lognormal distribution, thus establishing that only 5% of our participants achieved a $2^{\circ }$ mean absolute error and finding evidence suggesting a lower value for the MAA. With our protocol offering an alternative to classical setups, we conclude by discussing how the increased access offered by WebVR may open to the use of localisation experiments as medical and psychoacoustic tools.

## Results

**Figure 1:**
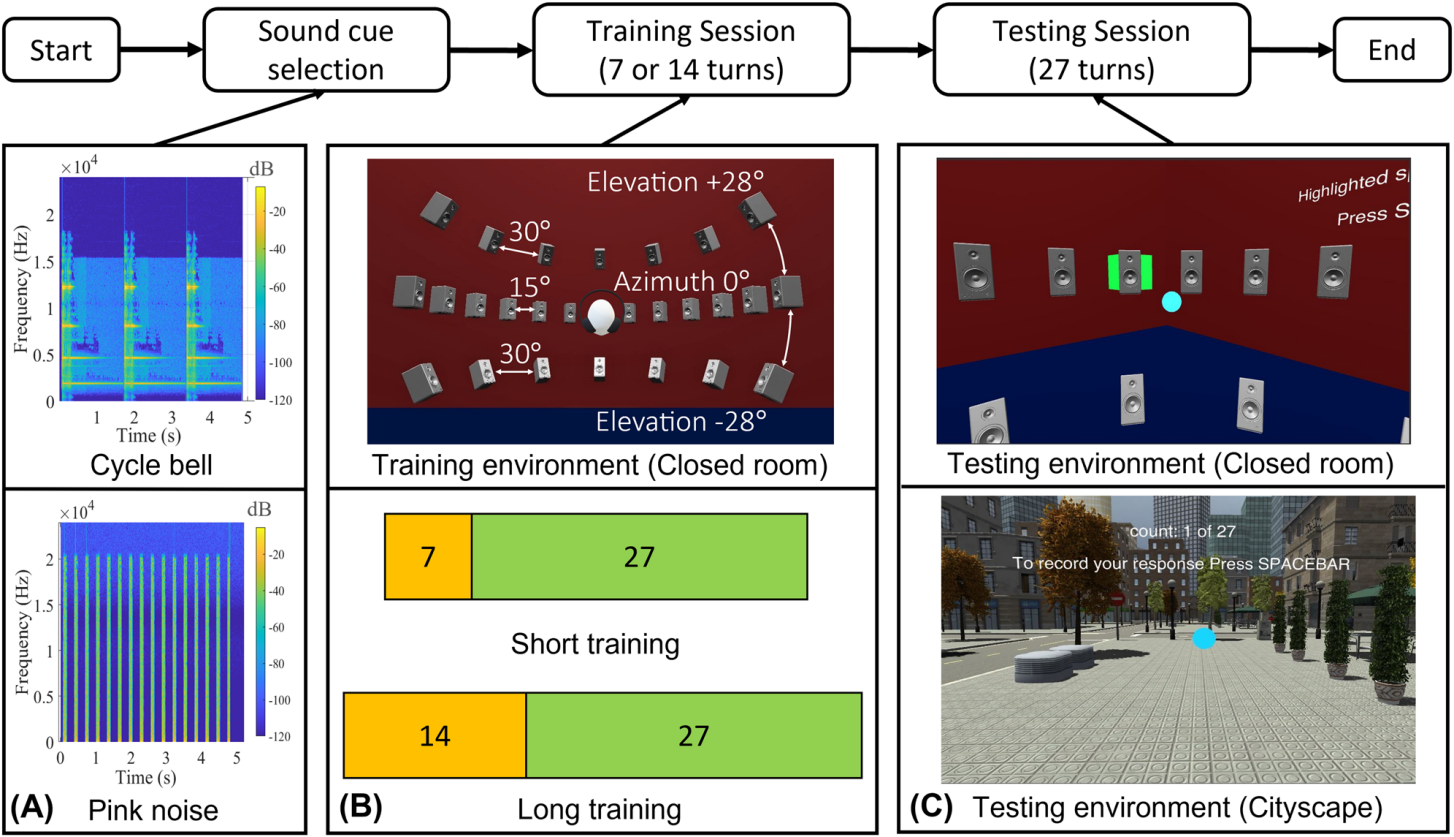
Illustration of the experimental design and detailed pipeline of the user study. At the top, the figure shows the workflow of the experiment. First, the user is randomly assigned a sound cue among two options: ‘Cycle bell’ and ‘Pink noise’. As shown in the spectrographs (**A**), Cycle bell (introduced by us, top of A) had tonal components below 1500 Hz, while Pink noise (the standard sound used in localisation experiments, bottom of A) was flat in frequency. Then, the user was brought into a ‘closed room’ for training purposes (**B**). The room (top of B) contained a total of 27 virtual sound sources: 13 visible sound sources in the azimuth plane (0$^{\circ }$ elevation) with an angular distance of 15$^{\circ }$  in each, and seven sound sources for each of the elevations of + 28$^{\circ }$  and − 28$^{\circ }$. The angular distance between the sources at non-zero elevation was 30$^{\circ }$. In the training room, we introduced two types of training (bottom of B): ‘short training’ with seven trials and ‘long training’ with fourteen trials. Finally, users were brought into a randomly selected testing environment (**C**) where they were asked to locate the source of 27 audio stimuli. We used two environments: a closed room (similar to the one used for training, with speakers well visible, top of C) and a cityscape environment (with no visual cues, the bottom of C).

In this work, we ran six online user studies, for a total of 210 participants, for a total of 5670 answers. All participants were 18 years old or above. All the six studies were approved by The Sciences & Technology Cross-Schools Research Ethics Committee (SCITEC) within the University of Sussex (application: ER/CR377/1) and were carried out in accordance with relevant guidelines and regulations (i.e., the UK Data Protection Act of June 2021 and the Helsinki declaration). In order to take part in the studies, participants were asked to sign an informed consent form. After providing informed consent, each of the participants followed a similar pattern, briefly detailed in Fig. 1 and described in detail in “Methods”.

Each participant experienced therefore a specific sound cue (pink noise or a cycle bell—see Fig. 1A), had a certain training length (‘short’ or ‘long’ see Fig. 1B) in a IVE mimicking an empty room, and was finally brought into one of the two available (virtual) testing environments (‘empty room’ or ‘cityscape’—see Fig. 1C) for the experiment. The experiment itself consisted of delivering the specific sound cue in 27 different positions (see the top of Fig. 1B). For each audio cue delivered, we asked the participant to locate it in a 3D environment, while we recorded the reported azimuth (horizontal angle) and elevation (vertical angle), with the origin (0$^{\circ }$ azimuth, 0$^{\circ }$ elevation) in front of the user at the start of the experiment (see Fig. 1C).

**Figure 2:**
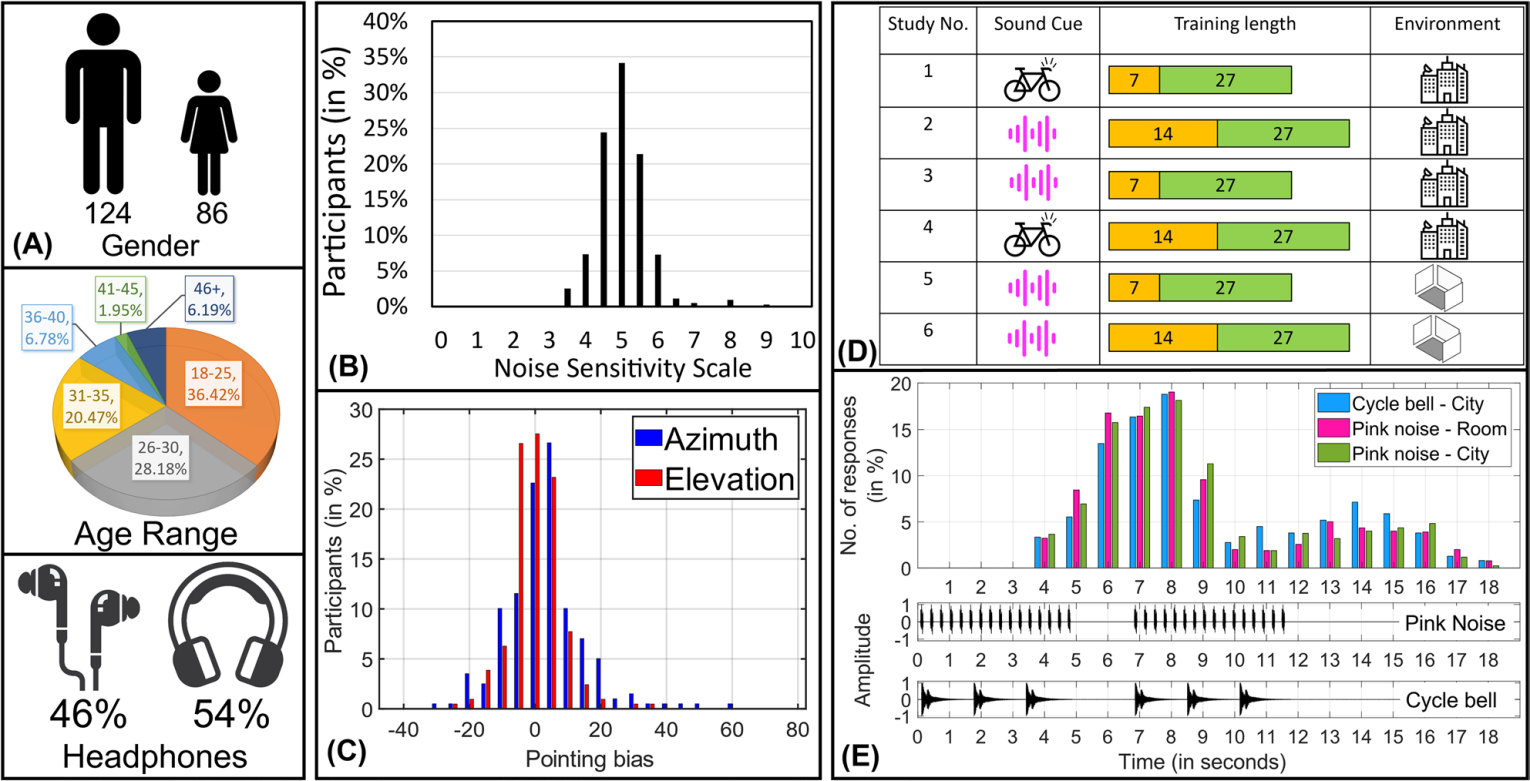
(**A**) (Top) Distribution of participants based on the gender by which they identified themselves. (Center) Distribution of participants based on their age range. (Bottom) Percentage of participants using in-ear and over-ear headphones. (**B**) Distribution of participants on the noise sensitivity scale from 0 to 10. (**C**) To minimize the error due to the fact that participants were new to WebVR, we calculated pointing bias. This is the representation of the number of participants and their pointing bias. (**D**) A graphical representation of all six studies based on their sound cues, training length, and testing environment. (**E**) (Top) Distribution of the frequency of responses for each second from 0 to 18 s. (Bottom) Representation of the sound cue spectra for pink noise and the cycle bell on the timescale from 0 to 18 s. (**A**) Includes parts purchased from iStock.com.

As summarized in Fig. 2A, our participants (86 females, 124 males) had an average age of $30\pm 8$ years. Figure 2A also shows that, when instructed to wear headphones, 46% of the respondents declared they were wearing earbuds (in-ear), while the others declared they were using over-ear devices. All participants self-reported normal hearing as well as normal or corrected vision. Sensitivity to noise was self-assessed through an established test^17^, and participants were normally distributed around the middle of the scale (i.e., average = $5.2 \pm 0.7$ see Fig. 2B).

### Pointing bias

Considering that participants might be new to the idea of WebVR, it was hypothesized that their responses could be affected by a pointing bias. Following Ahrens et al.^7^, who observed a similar effect in speaker-based localization experiments, we calculated the bias for each participant both in azimuth (horizontal angle) and elevation (vertical angle) as the mean of all responses in the testing session. Figure 2C shows that the pointing bias was normally distributed around 0, hinting that the largest number of participants had a small pointing bias. The calculated bias was then used to correct the individual responses for all conditions.

### Response time

Since the experiment was remotely conducted online and not in person, we expected some of the responses not to be of optimal quality^18^. As recommended in previous literature^19,20^, we, therefore, set a threshold for acceptance and used participants’ response time to reject outliers. As shown in Fig. 2D, we ran six different combinations of the parameters in Fig. 1. We used two sound stimuli (cycle bell and pink noise) to stimulate different parts of the signal processing in participants’ headphones (see “Methods”). As shown in Fig. 1A, each stimulus started at $t=0$ seconds and lasted approximately 5 s. As shown in Fig. 2E (bottom), each stimulus was repeated twice, with a gap of approximately 2 s between each repetition, for a total stimulation time of 12 s. Each participant was asked to locate the cursor (blue dot in Fig. 1D) where they thought the sound was coming from, with no limitation on time: i.e., they could also answer after the stimulus was finished.

Figure 2E shows the number of responses that were collected in each second of the experiment. The results are shown for the case of long training (i.e., a cycle bell as a sound cue in the city environment; pink noise as a sound cue in the room; pink noise as an acoustic stimulus in the city environment—see Fig. 2D) in the window 0 to 16 s. Figure 2E shows two Gaussian distributions: the largest is centered at 7 s (i.e., approximately 2 s after the first occurrence of the stimulus) and the other is centered at 14 s (i.e., 2 s after the end of the repeated stimulus). This result told us the average time that participants took to locate the stimulus and what the probability was that the task would take longer to be accomplished.

We, therefore, decided to ignore the answers which arrived too early (i.e., before 4 s from the start of the first stimulus) or too late (i.e., more than 4 s after the end of the second stimulus), thus excluding 698 answers (12%) from the database. It is worth noting that, in the case of short training, the excluded answers accounted for 15% of those available. The number decreased to 9.6% in the case of long training, hinting at a positive effect of training on accuracy.

### Sound localization accuracy

**Figure 3:**
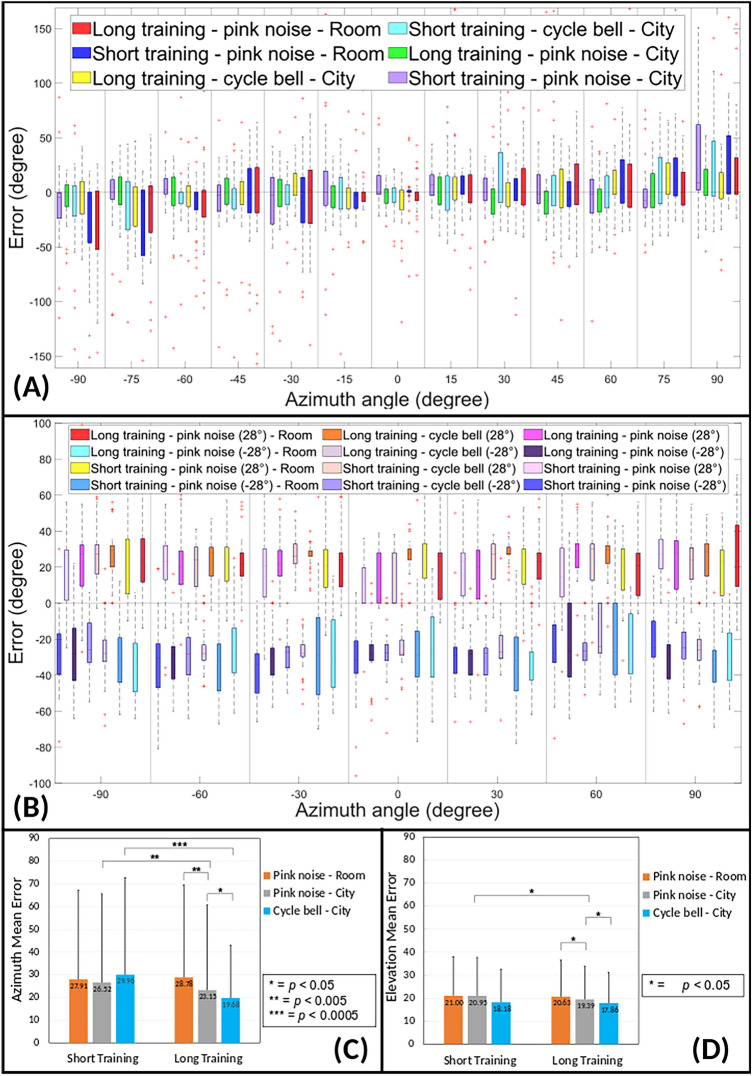
Box-plots showing errors in azimuth and elevation. (**A**) Angle-by-angle comparison of error observed in azimuth (horizontal plane). Includes all six studies together. ‘Error’ from the vertical axis is obtained by subtracting ‘response position’ from ‘sound cue position.’ (**B**) Angle by angle comparison of error observed in elevation (vertical plane) for + 28$^{\circ }$  and − 28$^{\circ }$. Includes all six studies together. ‘Error’ on the vertical axis is obtained by subtracting ‘response position’ from ‘sound cue position.’ (**C**) Comparison of azimuth mean error for all six conditions with the main distribution based on training, highlighting the statistical significance by comparing two conditions at a time with t-test statistical analysis. A single asterisk means the two variables are significantly different, a double-asterisk means the two variables are very significantly different, and three asterisks means the two variables are extremely significantly different. (**D**) Comparison of elevation mean error for all six studies grouped by training, highlighting the statistical significance by comparing two conditions at a time with t-test statistical analysis. A single asterisk means the two variables are significantly different.

Figure 3A shows the difference between the position (in terms of the azimuth angle ${\theta }$) of the sound source in the VE and the position reported by the participant, for elevations ${\varphi }$ = 0$^{\circ }$  and ${\varphi }$ = ± 28$^{\circ }$  (see Figs. S1 and  S2 for individual representations of azimuth errors for elevation ${\varphi }$ = 0$^{\circ }$  and ${\varphi }$ = ± 28$^{\circ }$  respectively). The results are reported as boxed plots for each of the six combinations explored in this study (see Fig. 2D), to represent the distributions of the answers. In this plot, an error of 0 means that the participants localized the sound with high accuracy in the azimuth direction. It is observed that, in the case of long training, the boxes representing city environment outcomes (with pink noise or with the cycle bell) are always approximately centred around the horizontal axis. Moreover, for long training, the overall accuracy does not significantly change as we move away from 0$^{\circ }$ azimuth (i.e., the position in front of the user at the start). In the case of short training, instead, the accuracy is at maximum in the centre and decreases as we move away from azimuth 0$^{\circ }$ towards ${\pm }90^{\circ }$. It can also be observed that, in Fig. 3A, there is a shift toward negative values on the left and towards positive on the right, i.e., there is a tendency of participants to perceive the sources at larger angles from the frontal direction, also observed by^7^.

In this study, we observed an overall mean absolute error of 25.8$^{\circ }$  in localizing the correct azimuth angle, which changed over the different cases. In Fig. 2D, for example, it is reduced to 24.3$^{\circ }$  when using long training, a cycle bell sound cue, and a city environment (study no. 4 in Fig. 2D).

To understand the results thoroughly, we calculated the absolute mean error for each of the six studies and organized the data into two main columns, i.e., short training vs long training (see Fig. 3C). In this way, each column had three sub-columns (each with 35 participants): (1) pink noise in the room, (2) pink noise in the city, (3) cycle bell in the city. To compare the results statistically, we used a two-sample (independent) *t*-test, displaying the sample means of each group, and their significance in Fig. 3C. We found that:

1. The overall difference between the mean absolute error obtained with long training (studies no. 2,4,6) and the mean error measured after short training (studies no. 1,3,5) is significant (*p*$<0.0005$).
2. In the case of long training, there is a significant difference (*p*$<0.05$) between the mean value measured with pink noise in the city environment (study no. 2) and that obtained with the cycle bell in the city environment (study no. 4).
3. The mean value relative to long training with pink noise in the city (study no. 2) shows a significant difference (*p*$<0.005$) to the one obtained with long training and pink noise in the room environment (study no. 6).
4. The mean value with long training and the cycle bell in the city (study no. 4) shows a significant difference, (*p*$<0.0005$) from the one with short training and cycle bell in a city environment (study no. 1).
5. For pink noise in the city environment, there is a significant difference (*p*$<0.05$) between the mean value obtained with long training (study no. 2) and short training (study no. 3).
6. In the room environment, however, we found no significant difference between pink noise with long training (study no. 6) and pink noise with short training (study no. 5).

In this study, we also looked at localization in the elevation (vertical) angle ${\varphi }$. Like for the azimuth, we used the whole set of sources: those at ${\varphi = 0^{\circ }}$ elevation (where the sources were spaced at ${\theta =-90^{\circ },-75^{\circ } \ldots +75^{\circ }, +90^{\circ }}$) and those at ${\varphi =\pm 28^{\circ }}$ (where the sources were at ${\theta =-90^{\circ },-60^{\circ } \ldots +60, +90}$). Figure 3B shows an angle-by-angle comparison of elevation errors for all six studies and $\varphi = \pm 28^{\circ }$. In general, we noticed that the values are not positioned around zero: the sources positioned at ${\varphi = +28^{\circ }}$ were perceived with a positive error (i.e., at higher angles), while those at ${\varphi = -28^{\circ }}$ were perceived with a negative error (i.e., at lower angles). We also noticed that not only does long training have higher accuracy than short training but that accuracy and precision degrade as we move further away from the centre. Figure 3B also shows that, compared to other conditions, long training with the cycle bell is consistent in precision within the azimuth range between − 30$^{\circ }$ and + 30$^{\circ }$ (see Fig. S3 in the supplemental for angle-by-angle comparison for elevation angle 0$^{\circ }$  which highlights similar observations as described above.) Our results, considering all the elevations, give an overall mean absolute error of 19.7$^{\circ }$  in elevation, which is reduced to 18.0$^{\circ }$  in study no. 4 (i.e., long training, cycle bell, and city environment).

As for azimuth, we calculated the mean absolute error for all the conditions and compared them in Fig. 3D. Also displayed in Fig. 3D is the statistical significance of the differences, obtained using a two-sample (independent) *t*-test. We can observe that:

1. In terms of the overall mean error, there is a significant difference between the conditions of long training (studies no. 2, 4, 6) and those of short training (studies no. 1, 3, 5), also in elevation (*p* $<0.05$).
2. For pink noise in the city environment, there is a significant difference between long training and short training (*p* $<0.05$).
3. Using long training, there is a significant difference between the mean error obtained with pink noise in the city and the one measured with pink noise in the room (*p* $<0.05$), which is again significantly different from the cycle bell in the city.

### Detailed statistical analysis

**Figure 4:**
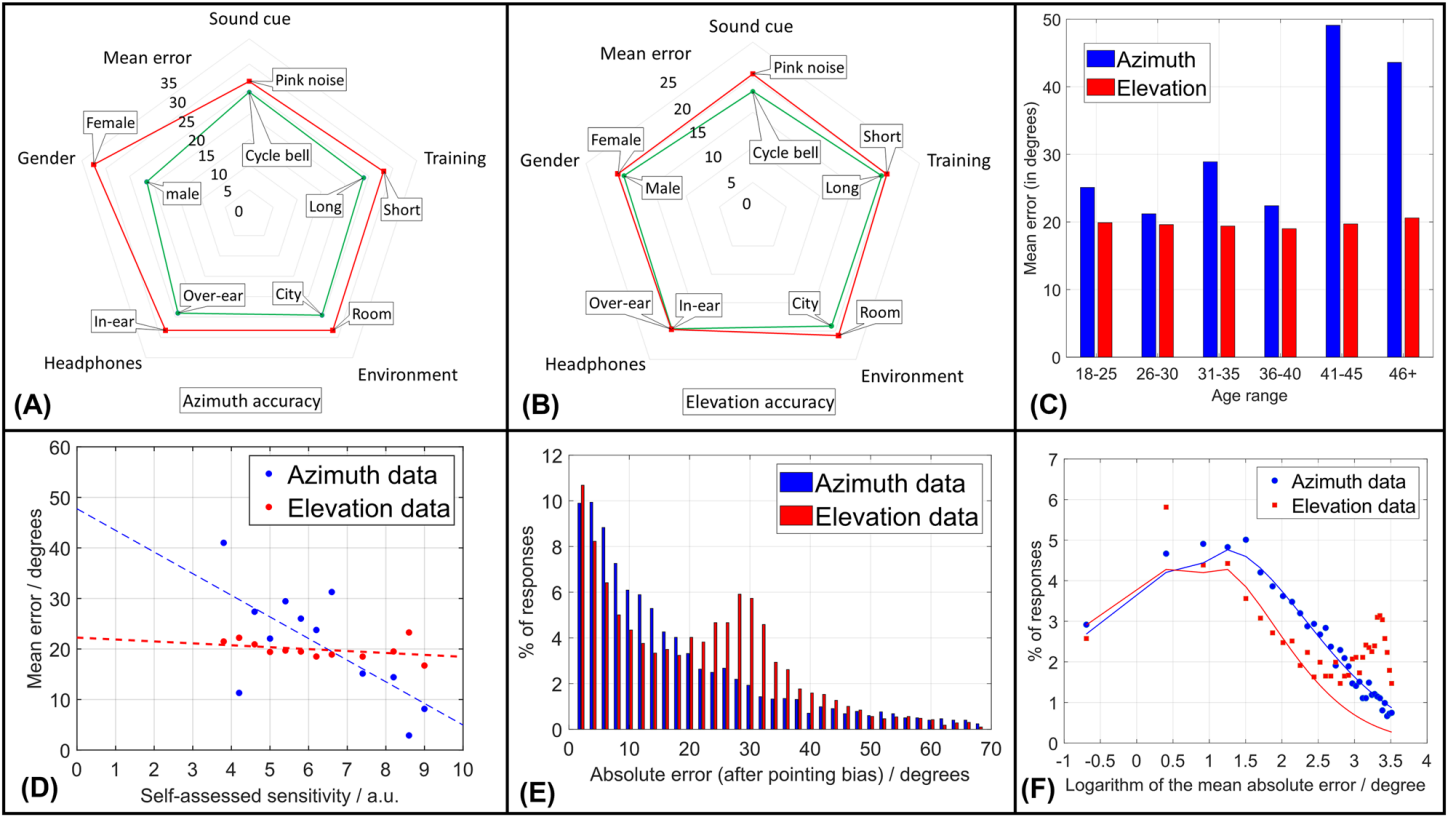
A graphical summary of the discussion. The radar plots (**A**,**B**) represent the mean absolute error obtained by varying only one of the binary parameters for azimuth (**A**) and elevation (**B**). For instance, by varying the training, the mean absolute error in azimuth changes from 30$^{\circ }$  to 24$^{\circ }$, and when comparing different genders, the mean absolute error changes from 33$^{\circ }$  to 17$^{\circ }$. The effects of the non-binary parameters are represented in (**C**) for age and in (**D**) for sensitivity. The overall distribution of the answers (for azimuth and elevation) is represented in (**E**) using a linear scale, to highlight the second peak in the answers for elevation. The same distributions of the answers are reported in (**F**) with the corresponding Log-Normal fits, for both azimuth and elevation.

In order to interpret the observations above, we conducted a statistical analysis using non-parametric tests. This was necessary since the data was not normally distributed (see Fig. 4E and next section).

We analyzed the data using the *Mann Whitney U Test*, which is the non-parametric version of an independent *t*-test. In the following, we report only the cases in which the comparison is statistically significant ($p<0.05$). As shown in Fig. 3, we observed that:

- there is an overall ‘training’ effect, significant for both azimuth and elevation;
- there is an overall effect of ‘environment’ on azimuth, for both long and short training;
- using pink noise (fixed-parameter), training has an effect on ‘elevation’;
- in the case of ‘long training’ (fixed-parameter):
  - ‘environment’ has an effect only on ‘azimuth’ while ‘sound’ has an effect on both ‘azimuth’ and ‘elevation’
  - keeping ‘environment’ as the fixed parameter, the cycle bell leads to lower errors than pink noise;
- ‘long training in city environment’ generally performed better as a condition for ‘azimuth.’

Using an analysis of variance test (ANOVA), we then looked for combined effects—for example, an interaction effect between environment and sound, the main effect of training time, or a main effect of sound.

We, therefore, sought a multi-linear correlation with the mean absolute error in azimuth (*Az*) as a dependent variable and the three parameters in Fig. 2D as independent binary variables, i.e., Environment, Training, and Sound. This procedure highlighted three correlations, respectively: negative for training (error decreases when training is long); negative for the environment (error decreases when in the city); and negative for sound (error decreases when the cycle bell is used). Of those, only the first two were statistically significant, with $F(2, 4969) = 12.85$, *p* $< 0.0001$ and $R^2 = 0.005$. This led to:

1 $$\begin{aligned} Az = 30.4 - 4.1\cdot (\mathrm {Training}) - 3.6 \cdot (\mathrm {Environment}) \end{aligned}$$

where both variables have a binary value (0, 1), with Training = 1 when Long and Environment = 1 when City. However, the overall probability that a multi-linear regression (with Training and Environment as binary values) explains the dispersion of our data for *Az* is 0.5%, which is low but significant. Further studies with more training times and environments will be needed to evaluate a better correlation.

We repeated the procedure for the mean absolute error in elevation (*El*) and, while there is evidence of a trend with Training and Environment similar to that observed for *Al*, according to ANOVA the most significant variable is ‘sound’, with $F(1, 4969) = 28.73$, *p* $< 0.001$ and $R^2 = 0.006$. This leads to the (statistically significant) regression:

2 $$\begin{aligned} El = 20.5 - 2.5\cdot (\mathrm {Sound}) \end{aligned}$$

where ‘sound’ has a binary value (0, 1) and Sound = 1 when using the cycle bell. The probability that this multi-linear regression model explains the variability in our *El* data is 0.6%, which is again significant but low. Further studies examining different sound stimuli will be needed to produce a better correlation.

As a final verification of our results, we calculated one mean error value for each participant and compared the results. We found that for ‘azimuth’, the mean error value for sound localization in ‘long training’ (24.9$^{\circ }$) was by 4$^{\circ }$ than in the case of ‘short training’ (28.9$^{\circ }$). For ‘elevation’, the mean error value for ‘long training’ (19.1$^{\circ }$) was approximately 1.2$^{\circ }$  lower than the one for ‘short training’ (20.3$^{\circ }$).

From the data collected through the post-experiment questionnaire, we observed an effect of headphones in ‘long training—cycle bell—city environment.’ Participants with ‘earbuds (in-ear)’ performed better in ‘elevation,’ but this effect was only visible in these conditions (i.e., study no. 4). We also observed a correlation with sensitivity (see Fig. 4B and next session). In ‘long training—pink noise—city environment’ for elevation ($p = 0.07$) and ‘long training—cycle bell—city environment’ for azimuth ($p = 0.08$), for instance, the localization error was clearly lower with higher sensitivity.

## Discussion

As reported by^10^, the concept of VR was formulated in the 1960s, with the first commercial VR tools appearing in the late 1980s. But it is in the last decade that this technology has risen in the public interest, propelled by lower accessibility costs, but also by Hollywood movies like *Ready Player One* and games like *Batman: Arkham VR*^21^. Even so, VR headsets are still not a widespread commodity: according to the latest estimates, only 171 million people used VR worldwide in 2019^22^. This is why WebVR is a valid alternative.

As described^23^, the main goal of WebVR is to offer VR to standard web browsers, thus simplifying access and enabling content to be shared more widely. WebVR is therefore very attractive to industry stakeholders, which makes this technology dynamic. Recent studies^24^, for instance, have found that, by using WebVR for learning, not only experience but also outcomes were enhanced. During periods in which it was not possible to travel and explore new places or meet people physically (e.g., while quarantined at home for disease prevention) VEs like Second Life^25^ and MelodyVR^26^ gained attention. In these environments, the correct delivery and localization of sound cues are crucial for interaction. Spatial audio has a strong impact on improving the sense of engagement with the virtual world^27^.

Recently, Steadman et al.^28^ found an effect of training on sound localization in VR after multiple 12-min training sessions delivered over multiple days. In our study, we did not limit the time of our training session, but in the ‘long training’ condition simply repeated the same training task twice, in a virtual room where speakers were visible. Assuming a maximum response time of 16 s (see Fig. 2E) for each of the seven speakers in the short training (see Fig. 1B) session, our training conditions were only 2 and 4 min long, but still sufficient to show a statistically significant effect. Further studies on this adaptation time may be crucial to determine how our brain adapts to immersive environments and how to design more accessible applications^14,29^.

We used two different sound cues (see “Methods”): one containing mainly frequencies below 1500 Hz (cycle bell) and the other (pink noise) classically used because it contains a broader range of frequencies (see Fig. 1A). This choice is due to the way sound is delivered through headphones^30^: lower frequencies rely on time and intensity differences between the left and right ear but do not depend on the listener’s head position, while higher frequencies rely on how the headphones deliver the ‘standard’ head related-transfer function (HRTF) e.g., the one pre-recorded into Unity 3D, which in our case, we assumed to be sufficient for our study^16,31^. We, therefore, expected a more accurate localization from the cycle bell (which we observed), but also a minimal effect of the headphones used (during the experiment) to deliver the sound, due to the use of a standardised HRTF^32^. We observed instead that participants using over-ear headphones had more accuracy for localization in azimuth. No significant difference was observed instead for localization in elevation (see Fig. 4A,B). This latter effect, however, was observed in only one case (i.e., study no. 4, which was the most accurate and therefore most sensitive to second-order effects). To explain why the cycle bell led systematically to more accurate responses, we, therefore, looked at the ‘environment.’

The two environments used in this study were visually very different in order to explore the potential effects of cross-modal perception^33^. After all, VR designers rely on the combination of both audio and visual cues to induce immersivity. Like in standard localization experiments, in the ‘room’ the speakers were visible and acted as clear spatial cues (see Fig. 1B). In the ‘city’, instead, the user was standing in a square with different visual stimuli, all disconnected from the audio ones (see Fig. 1C). Still, the results acquired in the ‘city’ environment were consistently more accurate than those obtained in the ‘room’ environment. Our results, therefore confirm that the presence of visual cues is not sufficient to give spatial information to the VR user, who instead needs movement^10,34^ or audio cues^3^. This result complements the findings by Kern et al.^35^, who studied the sense of ‘presence’ and ‘realism’ in VR by keeping the same VR environment but manipulating the presence of sound.

Furthermore, from the open comments at the end of the questionnaire, we realized not only that there was a congruence between the cycle bell sound and the ‘city environment’ but also that users found the cycle bell stimulus more familiar. This suggests that the stimuli played a role, intended as a ‘soundmark’^36^. We, therefore, believe that future localization experiments should consider different sound stimuli.

In our work, we created an immersive environment by combining the place illusion (PI, or ‘the feeling of being there’) and plausibility illusion (accepting that the events in the VE are really happening)^37^. Even though our participants knew that the environment was virtual, we, therefore, expected them to respond to it realistically^38^. Still, our mean average errors are much larger than those observed in ‘real’ experiments. As reported by^9^, in fact, the best results for the mean absolute error in azimuth obtained using virtual sources (i.e., sources programmed using the computer and delivered through headphones or loudspeakers) were, respectively, 12$^{\circ }$ using six virtual sources arranged horizontally, with sound delivered using headphones (21 participants, see^27^) and 2$^{\circ }$ with 13 virtual sources and sound delivered through loudspeakers (10 participants, see^7^). Since we used more than twice the number of participants required for statistical significance, given the number of parameters investigated (see “Methods”), our larger mean errors cannot be due to the larger number of participants/variables.

Figure 4A,B summarize the results of our studies for the binary variables. Each axis of the radar plots reports the mean absolute error obtained varying only one of the variables—for example, the mean absolute error in azimuth varies from 30$^{\circ }$ to 25$^{\circ }$ when changing environments, i.e., passing from the ‘room’ to the ‘city’. It can be clearly seen that while all the variables affect the mean average error in azimuth, there are very few changes in the elevation accuracy. The radar plots also show that there is an ‘ideal testing condition’ for azimuth, which leads to better accuracy.

If the binary variables highlight the link between audio and visual in WebVR, which potentially facilitates cross-modal perception^33^, our study highlights the role of other ‘non-auditory’ parameters on the accuracy of sound localization, often mentioned in soundscape research^39^.

In this sense, we explored two of the most commonly monitored parameters in soundscape research, often neglected in localization experiments: age of the participant (Fig. 4C) and self-assessed sensitivity (Fig. 4D)—see “Methods”. While we found no significant correlation between the mean absolute error and the age of the participants, Fig. 4C clearly shows that the older participants were less accurate. We explained this effect as a result of their inexperience with VEs, so further studies should examine whether extended training cancels this difference.

We found instead a clear parametrical correlation between the mean absolute error in azimuth and the self-assessed sensitivity (r= − 0.69 n =12, *p* = 0.014). The correlation can be expressed as

3 $$\begin{aligned} Az = 47.02 - 4.142 \cdot (\mathrm {Sensitivity}) \end{aligned}$$

where $(\mathrm {Sensitivity}) = 3 \ldots 9$ in our data. The linear regression in Fig. 4C has a 47% probability of explaining the variation in the mean absolute error in azimuth *Az*, with ANOVA $F (1, 10) = 8.9$. Unfortunately, due to the large dispersion of data, this correlation is not significant, with *p* = 0.014, and therefore needs further investigation. Understanding the effect of sensitivity on localisation tests may lead to a qualitative test to measure sensitivity to sound/noise (e.g., by comparing the result of a WebVR localization test to a calibrated sensitivity response).

Finally, we looked at the distribution of the answers. Figure 4E reports the overall distribution of the responses obtained in our studies, both for azimuth and for elevation. We notice that the distribution is non-Gaussian in both cases, with median absolute errors respectively at $\Delta _{\theta }=12^{\circ }$ for azimuth and $\Delta _{\varphi }=18^{\circ }$ for elevation. While the considerations made so far with the mean values are still relevant, the median better represents these types of distributions and, in the case of azimuth, the median absolute error agrees with the other VR results in the literature, which were found with a much smaller number of participants (and thus much fewer parameters).

The small variation found in passing to the median absolute error in elevation can be explained by the presence, in the distribution of Fig. 4E, of a secondary peak at approximately 28$^{\circ }$. Further analysis of the data showed that almost 16% of the participants did not perceive the sound had an elevation: they placed it at the correct azimuth $\theta$, but with $\varphi =0$. This difficulty in the vertical axis (elevation) was also observed in experiments correlating the perceived height of planes with their perceived noise^40^. In those experiments, observers tended to make a wrong assessment of the altitude of passing aircraft and thus alter their acoustic judgment. Further studies on this uncertainty in position sources in elevation may help to explain the large variability of annoyance statements due to aircraft noise in comparison to what is reported with other transportation sources for the same acoustic level^40,41^.

Having neglected the spurious responses for elevation, we fitted the data in Fig. 4E with a lognormal distribution with excellent results (see Fig. 4F). According to recent studies, in fact, this type of distribution better describes the response to acoustic stimuli^42^. The fit gives as most probable an angle of $2.4^{\circ } \pm 0.2$ in azimuth and $1.8 \pm 0.2$ in elevation. These values are very close to the physiological limit given by the minimum audible angle (*M*AA see^9^): a value that, according to our data, is only achieved by 5% of the population. The fact that we observed smaller mean absolute errors also hints at a potential lower value of the MAA.

This is a crucial insight for WebVR designers: it is certainly possible to space the sources in azimuth by 4$^{\circ }$, to capture people perceiving differences in location at the MAA level, but the associated cost in computational time will only give an effect perceived by no more than 10% of the users. Based on our results, instead, designing a virtual experience with sources spaced by 10$^{\circ }$  in azimuth (our median) would be sufficient to cover the localization ability of 50% of the end-users, for 1/6th of the computational time.

In summary, having considered all the outcomes and the non-auditory factors, our fully online experiment in WebVR provided complimentary insight to in-person sound localization experiments. Future studies will explore the role of adding more parameters as visual cues and in changing the virtual environment.

One of the key assumptions underpinning our results is that all the participants in our online studies used headphones. On the other hand, since it is not uncommon that online participants do not follow instructions, the difference between our average localisation error and the values found in the literature^9^ may be due to some participants using loudspeakers instead. This risk was reported by Milne et al.^43^, who used dichothic pitch to make sure their participants were using headphones and run a control study in supervised conditions (i.e. in person, with one of the researchers present). In our case, we mitigated this uncertainty at design stage: A) by adding (as suggested by Prolific) appropriate questions to check that the experiment was being run correctly and that the participants were paying attention; B) by making sure that our listening tests were difficult to run without headphones (this was confirmed in a pilot not described here). In addition, we quantified the potential impact of this uncertainty on our results by exploring the effect of the listening conditions on a selected listening test (“long” training, “cycle” sounds, “city” environment). Supplemental Fig. S4 shows a comparison between the online unsupervised results, obtained assuming that all the participants wore headphones, with two other conditions. The first is also an online experiment, where 35 randomly-selected participants were asked to use their computer’s loudspeakers to complete the listening test, without any supervision. The second is very similar to the typical in-person listening experiment from the literature, where 35 participants (selected among university colleagues) were tasked to complete the listening test in a controlled environment (50% used a fixed set-up), while supervised by one of the authors. The significant statistical differences between the three distributions (Fig. S4) support our original assumption that only a negligible number of participants did not wear headphones. They also support one of our conclusions, where we suggested that a low value of the average localisation error can be achieved by pre-selecting the listeners, but may not be fully representative of the real population (see Fig. 4).

Another key aspect to evaluate our results is their ecological validity. As reported in Xu et al.^44^, this parameter “describes the degree to which results obtained in a controlled experiment are related to those obtained in the real world”. To maximise this aspect, (1) we designed our experiment taking inspiration from studies which had found the same localisation error using a dome of speakers in the “real” and in the virtual world^7^; (2) we targeted the number of participants to a statistical power of 90%, to make sure that even smaller effects were captured and minimise false results (i.e. 10% probability of encountering a false negative result and 5% likelihood of encountering a false-positive result for the standard value for the significance level); 3) the interaction of participants with the experiment was controlled (i.e. since they had to point the cursor towards the perceived source, it was similar to asking them to keep their head still); 4) we asked participants to evaluate the immersivity of their experience, separately for visual and for audio, and added an open box to collect generic feedback (see supplemental). All these results point to an ecological validity above average (mostly above 4 on our 0–10 scale, depending on the virtual setting—see supplemental Fig. S5). No further indication on immersivity came from the visual analysis of the open responses at the end of the post-experiment questionnaire (see Fig. S6). According to Xu et al.^44^, a higher ecological validity could be achieved by using head-mounted displays and Ambisonics playback. Having found only a minor effect of the type of headphones worn by the participants (see Fig. 2) and a statistical difference between the online unsupervised results and the ones obtained with highly trained participants, we think that—in our case—ecological validity could be improved by an even larger data set. In future work, if our findings are confirmed (e.g. through a larger data set of participants), they may explain further the correlation between the number of speakers and average localization accuracy observed in previous studies^9^. They may also lead to immersive experiences with fewer speakers or with innovative ways of delivering localised sound^45^.

We can envision future applications for our study targeting sound localization difficulties and diseases^46^. Indeed, not only audio-related syndromes cause the loss of auditory spatial navigation. There are pieces of evidence of co-morbidity between localization difficulties and non-audio-related diseases. For example, researchers have observed this in people with diabetes^47^ or ischemic^48^ cerebral stroke. It would be interesting to explore the WebVR platform as a tool to help in the recovery and rehabilitation of such malfunctions. The WebVR platform is not only affordable and easily shareable, but it also gives opportunities to tailor environments, acoustic stimuli, and duration of the study to the end-user. In the long term, we see sound localization becoming an auditory test as normal as checking lateral sight at an optician.

## Methods

In this study, we ran a sound localization experiment using a WebVR platform. Typically, in sound localization experiments with real sound sources (loudspeakers), participants are asked to move their head and align it in the direction of the sound cue. Once the participants are confident about the direction of their heads they are asked to record their responses^49^, either by pressing a button or by responding verbally. In VR, this type of experiment occurs with virtual sound stimuli (headphones)^50^; participants are subjected to virtual sound stimuli coming from different directions and are asked to point in the direction of the sound source as they perceive it in the virtual world. In the virtual world, the design of similar experiments consists of three parts: the selection of sound stimuli, the choice of the environments to use as visual stimuli, and the procedure to follow. Considering these factors we designed our experiment in Unity 3D and compiled it in HTML5 format. We uploaded this compiled file onto an online platform (http://itch.io/) which could be accessed using any web-browser only with a unique URL. Only after we successfully received their consent to participate was this URL revealed to participants. To participate in this study, the use of headphones (in-ear or over-ear) was mandatory.

### Sound stimuli

For this study, we chose two types of acoustic stimuli: pink noise and a set of cycle bells. ‘Pink noise’ is a standard choice for these experiments, as it covers a large bandwidth. For easier bench-marking with literature, we followed^8^ and used a train of 16 pulses, each with a duration of 100 ms and a separation of 300 ms, for a total duration of 5000 ms. This type of stimulus is more congruent with the closed VR room used during testing (see below). For each selected location, the train of pulses was repeated twice, with an interval of 2000 ms between them.

The cycle bell was a non-standard source, but easily recognizable and congruent with the open VR cityscape used during testing. In terms of frequency content, this stimulus was still broadband, but with tonal components below 1500 Hz. Each cycle bell is comprised of three bell sounds, each lasting 1500 ms, for a total duration of 5000 ms. Two-cycle bells were used for each location (see Fig. 1).

### Sound source arrangement

To deliver the sound in a systematic manner, we designed a hemispheric speaker dome in Unity 3D. This structure is inspired by the work of^7^, where accurate spatial auditory perception was achieved using VR and loudspeakers with pink noise as a sound cue. In the speaker dome, there were a total of 27 speakers. In the horizontal plane (azimuth), 13 speakers were placed at an angle of 15$^{\circ }$  of separation. In the vertical axis (elevation), seven speakers were placed above the horizontal axis and seven more were placed below, with an angular separation of ${\pm } 28^{\circ }$. Horizontal spacing between each of those speakers was 30$^{\circ }$. We kept this structure consistent throughout the experiment.

#### Training session

Our experiment starts with the training session. The main goal of the training was to make participants aware of the experiment and enable them to become used to the sound stimuli as well as sound localization. For training, we selected seven sound source positions out of 27. In the horizontal axis (azimuth): − 60$^{\circ }$, 0$^{\circ }$, and 30$^{\circ }$; at + 28$^{\circ }$ on the vertical axis (elevation): − 90$^{\circ }$ and 60$^{\circ }$ ; at − 28$^{\circ }$ elevation: − 30$^{\circ }$ and 90$^{\circ }$. We designed two types of training, i.e., short training and long training. In short training, we delivered sound from seven different positions (as mentioned above). In long training, we repeated those seven positions twice (a total of 14 trials). Throughout the training, each turn started with a countdown. At the beginning of each turn, the pointer position was reset to one direction, meaning that every turn started from the same angle. During the training, participants saw a speaker dome as a visual cue. Once they confirmed their answer by moving the pointer towards the direction from which they thought the sound was coming and pressing the space-bar, a green box popped up at the correct sound source, highlighting the visual speaker and actual location of the sound source. This was to help participants realize how much they had misjudged the position of the source or if their response was accurate.

#### Testing session

Once participants finished their training session (long or short), they moved to the next phase, i.e., the testing session. During the testing session, we used all the source positions (i.e., a total of 27), delivered in random order. Here, participants did not receive any visual cues for the sound source. They were expected to judge the direction of the sound just by listening to the sound cue. Similar to the training session, for each turn, the pointer position was reset to the same angle and each turn started with a counter. After the sound cue was delivered, the participants were asked to confirm their answer by moving the pointer in the direction from which they thought the sound was coming and then pressing the space-bar. Unlike with training, they did not receive any feedback on their responses, but the session continued to the next turn. After finishing 27 turns, the testing session ended and so did the experiment. A questionnaire on sound sensitivity was used at the end of the experiment^51^. In the same questionnaire, we asked about the type of headphones participants used for the experiment. They had three options: (1) In-ear, (2) Over-ear, (3) No headphones. As a check on the participant’s involvement and to avoid tests run using smartphones, we also asked about the Operating System (OS) they used for the experiment, with the options: (1) Windows, (2) Mac, (3) Linux, (4) Other.

### Visual environments

For this study, we considered two types of visual environments. First, an empty room, and second, a cityscape.

#### Empty room

The empty room mimics the anechoic rooms typically used in these experiments^7,52^: it consisted of a floor, surrounding walls, and the speaker dome. The speakers were clearly visible in this environment. The placement of those visual speakers in the dome was the same as explained in “Sound source arrangement” section. To limit the visual distractions, we did not introduce any other visual cues to the empty room.

#### City environment

This environment was designed based on a package from the Unity asset store^53^. The city environment mainly consisted of buildings, roads, shops, trees, and a few vehicles. In this environment, we also used the same sound source arrangement described in “Sound source arrangement” section, but we did not show the visual speakers.

#### Pointer

In this WebVR experiment we used a ‘blue circle’ as a pointer (mouse pointer) to look around and indicate the perceived direction of virtual sound stimuli (Fig. 1B). We recommended participants use a mouse to move the pointer. To record the responses throughout the experiment, participants had to press the space-bar. Since WebVR has no head-mounted display (HMD), participants had to move the mouse to look around and keep their heads steadily pointed toward the screen. The movement of the mouse was considered as the movement of their head, i.e., first-person camera^54^. And the audio listener was attached to the camera, so when participants moved the pointer, the direction of the audio listener also changed automatically. This mechanism was used to render and understand the direction of the sound.

### Procedure

After giving consent to volunteer for the study through the consent form at the beginning of the experiment, each participant received a unique identifier (ID), which was required to start the experiment. As described in Fig. 1 (pipeline of the experiment), the experiment started with an introduction screen explaining how to proceed and what to expect during the experiment. Once the space-bar was pressed, the participant was led into the empty room for a training session (Fig. 1B). Once this was over, the scenario changed to the testing session (Fig. 1C).

### User studies

We estimated the required number of participants *N* by running an a-priori statistical power analysis for sample size estimation in G*Power^55^. Running a power analysis (using IBM SPSS Statistics-Version 26 software) on a repeated-measures ANOVA with two sound conditions (i.e., cycle bell, pink noise), a power of 0.90, an alpha level of 0.05, and a medium effect size (f = 0.25, critical F = 4.11), gave the desired sample size of approximately $N=45$ participants. Adding the training condition as a further parameter (i.e. ‘short training’ and ‘long training’) increased the desired number of participants to $N=80$. Given the difficulties related to interpreting online surveys, we decided to over-sample and recruited 210 total participants ($N= 35$ for each study) through an online crowd-sourcing portal (https://prolific.co/). Participants received a link (URL) with instructions, a consent form, and a unique identification number, which had been generated automatically. We used the experiment set-up above in six different user studies. Key variables which we changed during these six studies were sound cues and length of training turns. In *Study 1: Cycle bell, short training*, the cycle bell was used as the audio stimulus, which was more congruent with the city environment, but mostly contained frequencies below 1500 Hz. In *Study 2: Pink noise, long training*, the sound stimulus was pink noise, but the training time was doubled. It also occurred in the city environment. In *Study 3: Pink noise, short training*, which was intended as a benchmark with the literature, we used the same conditions as in^8^. The city environment was used for testing. In *Study 4: Cycle bell, long training*, the sound stimulus was the cycle bell, but the training time was doubled. The environment used for testing was ‘city.’ For the last two studies, in order to understand the impact of visual cues, we conducted testing in a ‘room’ environment. *Study 5: Pink noise, short training*, sound stimuli, and experiment length were the same as Study 3, but training, as well as testing, happened in the ‘room’ instead of the ‘city’ environment. In *Study 6: Pink noise, long training*, we repeated the same visual conditions as the previous study but doubled the training time.

## Supplementary Information

Supplementary Figures.

Supplementary Video 1.

## Data Availability

The data that support the plots within this paper and other findings of this study are available from the corresponding author upon reasonable request.
