# Supplementary material for: Sound localization in web-based 3D environments
Source: Sci Rep. 2022 Jul 15;12:12107. doi: 10.1038/s41598-022-15931-y (PMC9287443; doi:10.1038/s41598-022-15931-y)
Supplement: Supplementary file 1 — Supplementary Figures. [file 41598_2022_15931_MOESM1_ESM.pdf]

Supplementary Materials

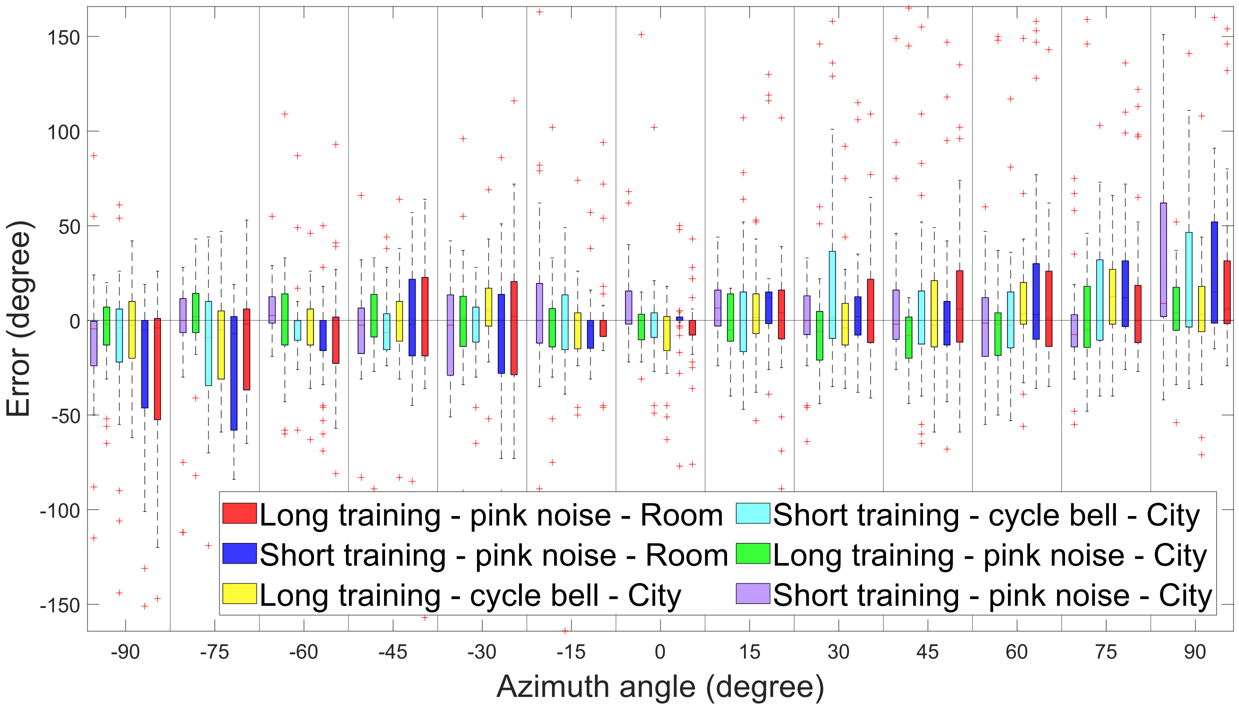

Figure S1: Boxplot showing an angle-by-angle comparison of localization errors observed in azimuth, relative to elevation angle 0° (horizontal plane). This figure represents a sub-set of the data in Figure 3A and includes all the six studies together. The 'Localisation error' in the vertical axis is obtained by subtracting 'response position' from 'sound cue position.' It is observed that localization accuracy for long training with a cycle bell in a city environment does not change much as we move further away from 0° while short training shows a reduction in accuracy as we move away from 0°.

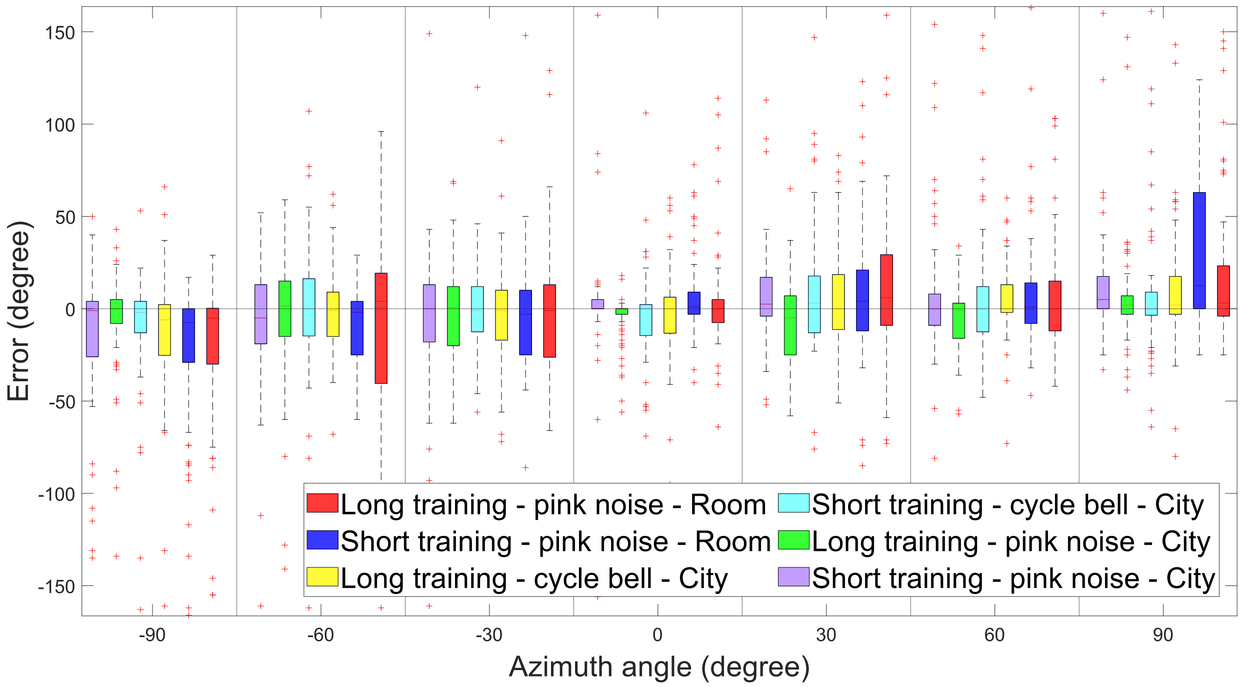

Figure S2: Boxplot showing an angle-by-angle comparison of the localisation errors observed in azimuth (horizontal direction, from left to right), but relative to the elevation angles +28°, and -28° together. This figure represents a sub-set of the data in Figure 3A and includes all the six studies together but highlights that there are fewer azimuth angles at elevation angles +28°, and -28°. 'Localisation error' in the vertical axis is obtained by subtracting 'response position' from 'sound cue position.' It is observed that the overall performance of participants after long training is consistent for all angles. This is not true for short training.

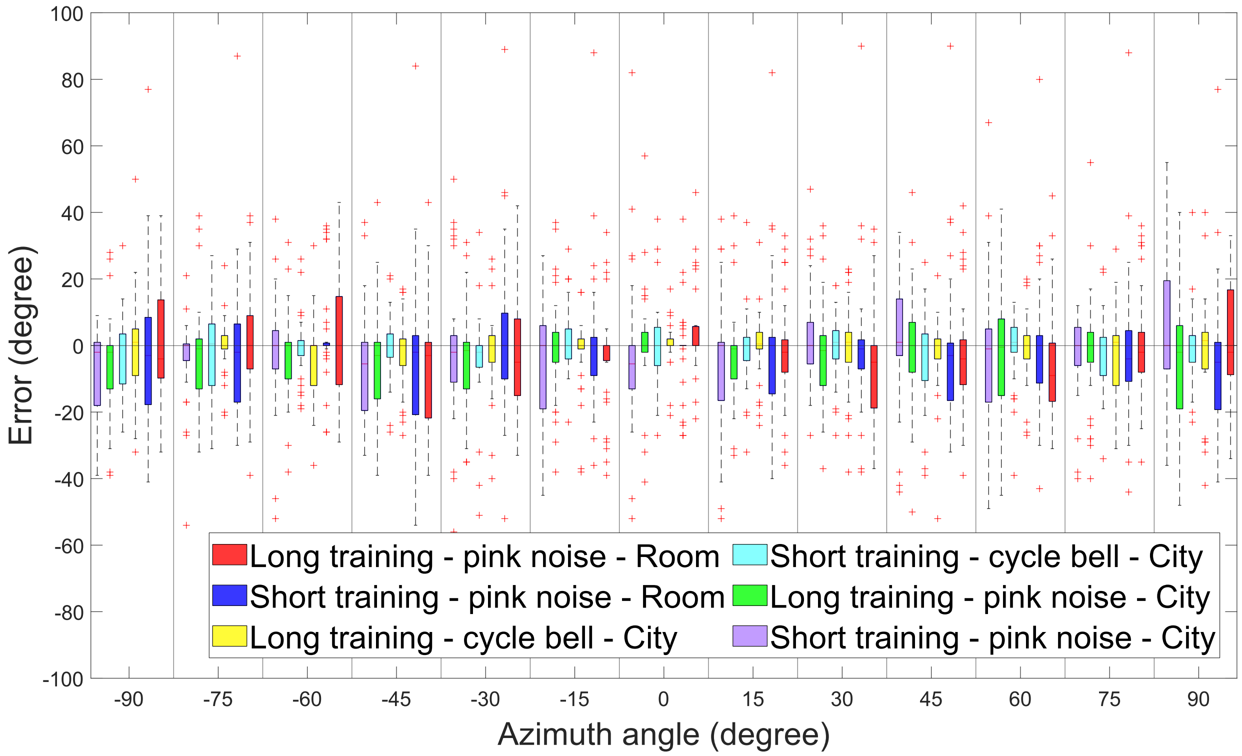

Figure S3: Boxplot showing an angle-by-angle comparison of localisation errors observed in elevation (vertical direction), relative to elevation angle 0°. These data have not been shown in Figure 3B but are considered in the analysis (from 3D onwards). They include all the six studies together. 'Localisation Error' in the vertical axis is obtained by subtracting 'response position' from 'sound cue position.' It is observed that the cycle bell in the city environment shows a consistent performance throughout all the angles, especially for long training. Pink noise, conversely, shows a minimum error at 0° and an increased error when the angle moves towards -90° and +90°.

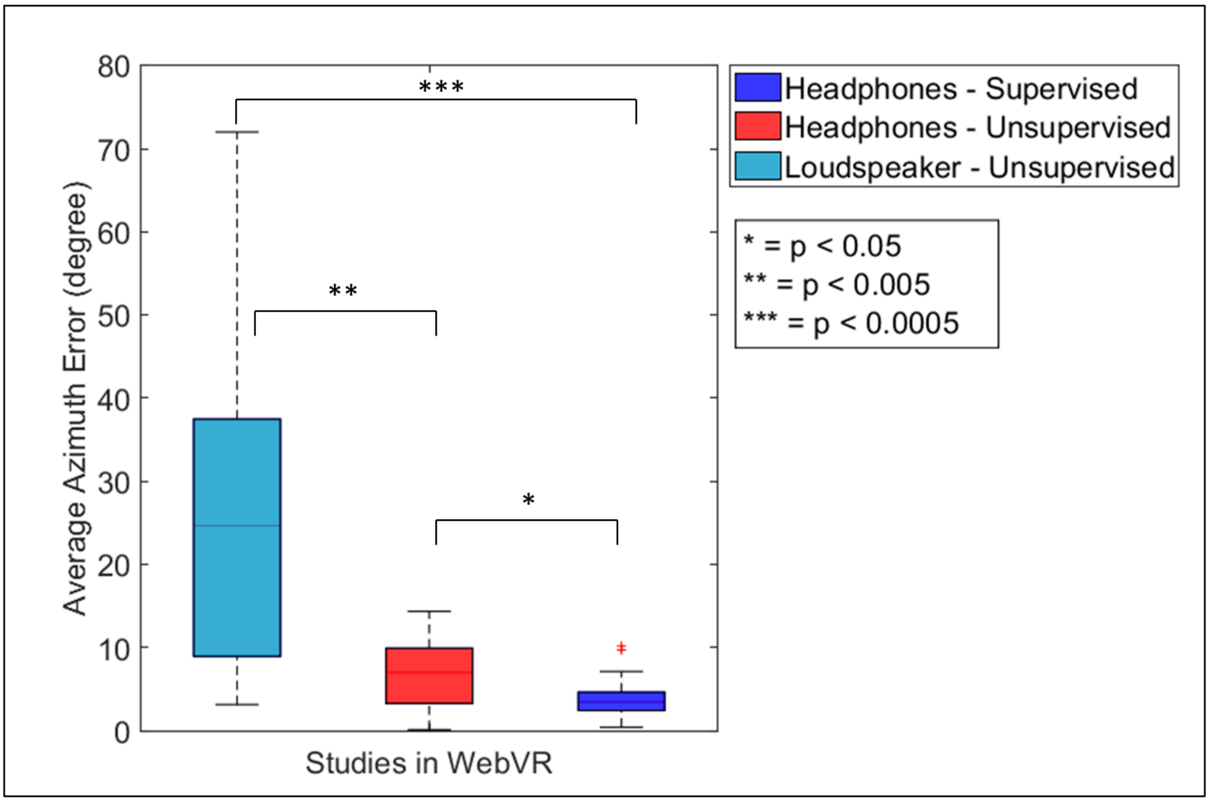

Figure S4: Bar chart comparing the distribution of localisation errors for the same experiment ("long" training, using a "cycle bell" within the "city environment") run in three listening conditions (each with 35 participants). For the first condition – 'loudspeaker, unsupervised' – participants were recruited online and conducted the experiment unsupervised using their computer's loudspeakers (i.e. minimal control on the listening conditions). The second case, which we labelled 'headphones - unsupervised', refers to one of the cases discussed elsewhere in this study. For the last condition ('headphones - supervised') the participants were recruited in-person – among people who were aware of the research – and conducted the experiment using WebVR, but with one of the researchers in the room. All of the participants in 'headphones – supervised' were wearing headphones and 50% of them used the same set-up. The comparison shows that the condition 'loudspeaker-unsupervised' is significantly different from both the fully supervised one ($p<0.0005$) and the one run for prolific in this study ($p<0.005$). The difference between the two listening conditions using "headphones" ($p<0.05$) is probably due to the fact that a single set-up was used. The comparison also supports the hypothesis that only a negligible number of users, if any, did not wear headphones in this study, while obtaining the results in Figures 3 and 4.

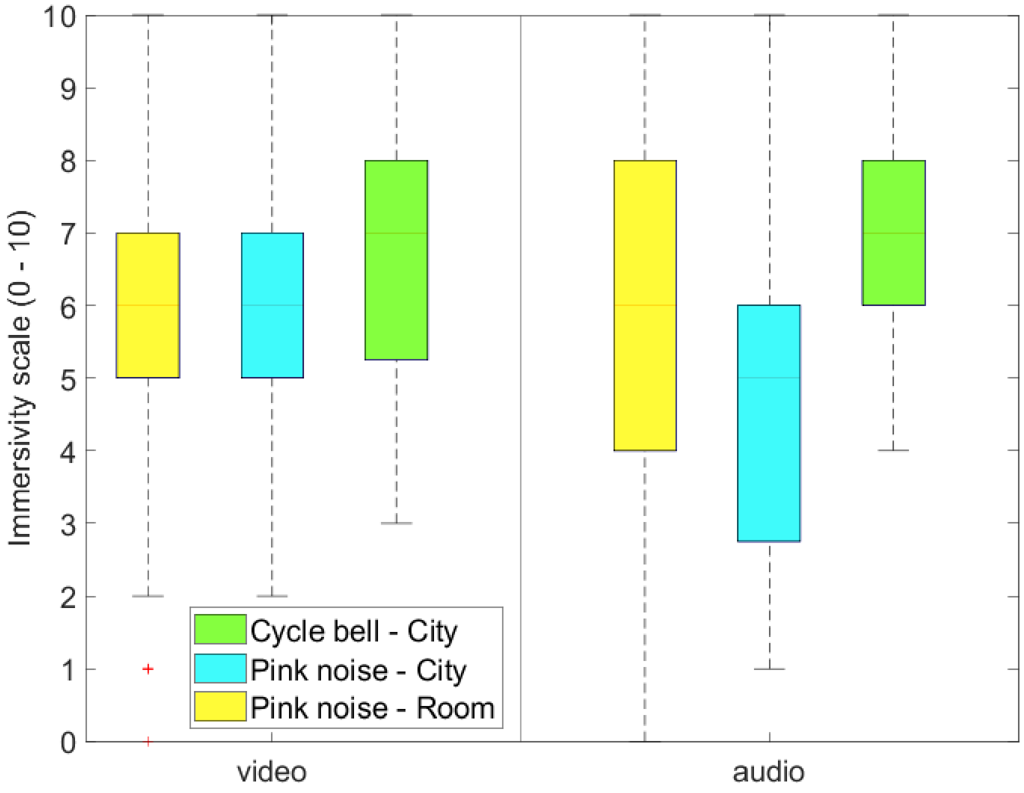

Figure S5: Box plot showing the distribution of the immersivity responses for the two parameters: Visual and Audio. This illustration is based on the data collected from the questionnaire post-experiment. We asked participants how immersive the visual appearance and audio element in the testing environment was on a scale of 0 to 10. This data includes results from all six studies. We have combined results for long training and short training for each of the cases: ‘pink noise – room’, ‘pink noise – city’, and ‘cycle bell – city’. While all the values are above the middle of the scale, it is observed that ‘cycle bell – city’ has been perceived with higher immersivity for both visual and audio elements while ‘pink noise – city’ has the larger percentage of lower scores.

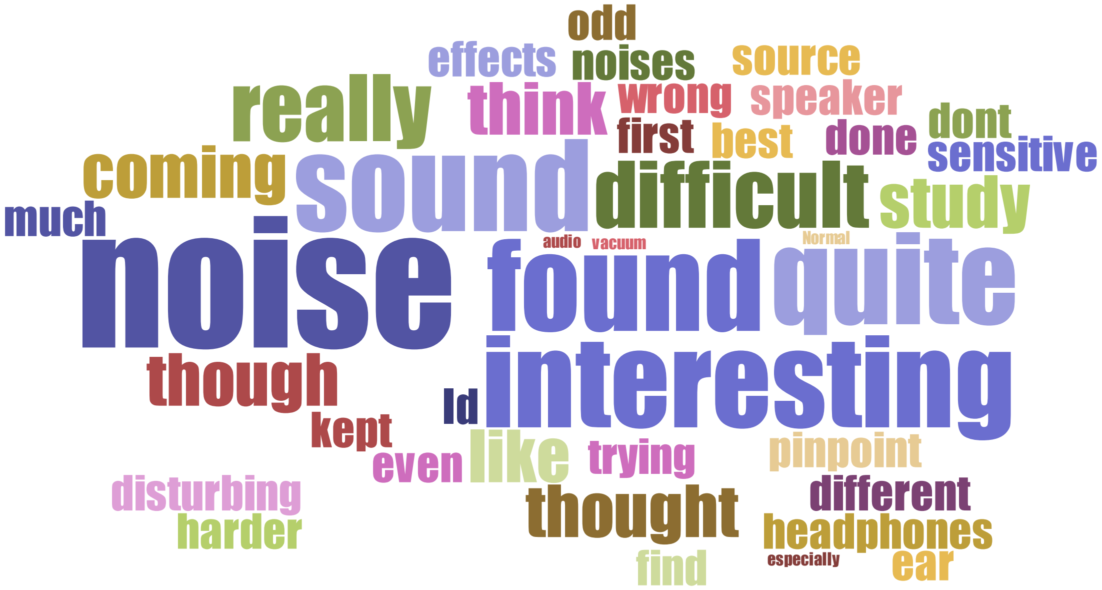

Figure S6: Word cloud showing the highest used words by the participants to describe their experience of this study. This illustration is based on the data collected from the questionnaire post-experiment. We asked participants what they think about the study as an optional question with a textbox. This data includes results from all six studies.
